# Plasmonic Nanofluids: Enhancing Photothermal Gradients toward Liquid Robots

**DOI:** 10.1021/acsami.3c06859

**Published:** 2023-10-18

**Authors:** Matteo Bevione, Alessandro Chiolerio, Giulia Tagliabue

**Affiliations:** † Empa—Swiss Federal Laboratories for Materials Science and Technology, Ueberlandstrasse 129, 8600 Duebendorf, Switzerland; ‡ Center for Converging Technnologies, Soft Bioinspired Robotics, Istituto Italiano di Tecnologia, Via Morego 30, 16163 Genova, Italy; § Laboratory of Nanoscience for Energy Technology (LNET), École Polytechnique Fédérale de Lausanne, Rte Cantonale, 1015 Lausanne, Switzerland

**Keywords:** Titanium nitride, Photothermal conversion, Mie theory, Heat transport in nanofluids, Energy harvesting

## Abstract

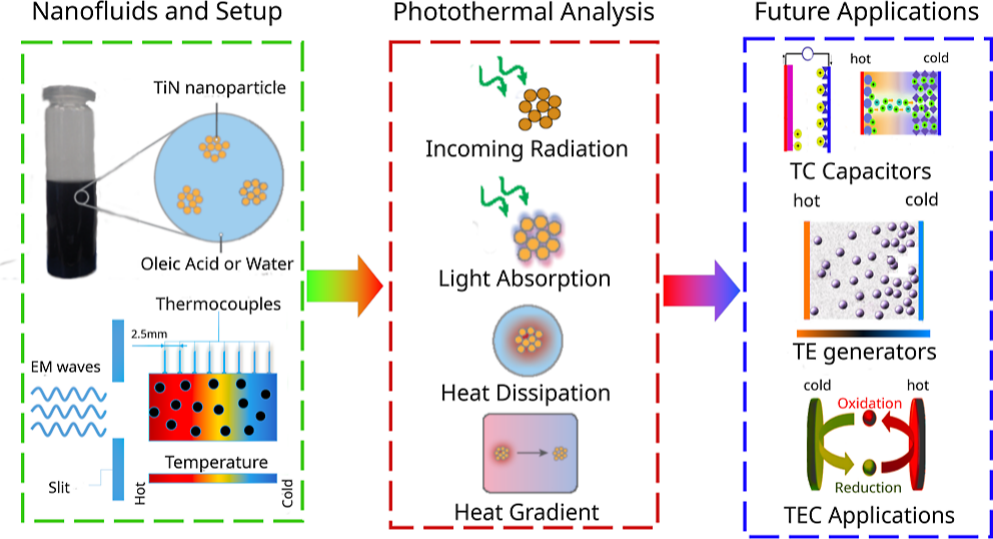

In situ energy generation in soft, flexible, autonomous devices is challenging due to the need for highly stretchable and fault-resistant components. Nanofluids with pyro-, tribo-, or thermoelectric properties have recently emerged as promising solutions for realizing liquid-based energy harvesters. Yet, large thermal gradients are required for the efficient performance of these systems. In this work, we show that oil-based plasmonic nanofluids uniquely combine high photothermal efficiency with strong heat localization. In particular, we report that oleic acid-based nanofluids containing TiN nanoclusters (0.3 wt %) exhibit 89% photothermal efficiency and can realize thermal gradients as large as 15.5 K/cm under solar irradiation. We experimentally and numerically investigate the photothermal behavior of the nanofluid as a function of solid fraction concentration and irradiation wavelength, clarifying the interplay of thermal and optical properties and demonstrating a dramatic improvement compared with water-based nanofluids. Overall, these results open unprecedented opportunities for the development of liquid-based energy generation systems for soft, stand-alone devices.

## Introduction

Nanoparticle-seeded fluids, also called nanofluids, uniquely combine the advantages of liquid systems, such as efficient advection-based energy transport and shape-adaptability, with tunable thermal transport properties (i.e., conductivity and diffusivity).^1,2^ This dual nature makes them appealing for a variety of applications, from heat transfer to energy harnessing, generation, and storage.^3^ In particular, nanofluids offer unmatched reliability for flexible devices and liquid/soft robotics, where highly stretchable and fault-resistant components are critically needed. Moreover, by incorporating nanoparticles with magnetic as well as pyro-, tribo-, and thermo-electric properties, they offer new opportunities to realize liquid-based stand-alone harvesters that can recover energy from the environment. For example, under the application of a thermal gradient, ferrofluids can generate an electromotive force,^4^ while water-based barium titanate and titania nanofluids have shown sizable power generation by pyro- and triboelectric mechanisms.^5,6^ Moreover, the addition of thermoelectric nanoparticles, such as Bi_0.4_Sb_1.6_Te_3_, to a water-based electrolyte greatly improved the power output of a thermogalvanic cell.^7,8^

Interestingly, the synergistic use of photothermal effects in nanofluids, that is, conversion of electromagnetic radiation into heat through photon absorption, could augment and sustain electrical power generation by the mechanisms described earlier. In fact, while the liquid phase is typically transparent in the visible spectral range, the solid phase can be engineered to effectively absorb incoming (solar) radiation.^9,10^ Consequently, the dissipation of photon energy into heat within the particles is subsequently transferred to the carrier fluid, resulting in the overall heating of the nanofluid.^11,12^ These features have found promising applications in solar thermal processes, such as solar-driven water purification or desalination.^13^ Recent works have focused on the use of carbon black^14^ and core/shell materials^15^ to enhance absorption and photothermal efficiency. Similarly, nanofluids can be enhanced with energy generation capabilities by incorporating a solid fraction with pyro-, tribo-, or thermo-electric properties.^16^ Plasmonic (metallic) nanoparticles, exhibiting highly absorbing and tunable optical resonances, have recently emerged as effective photothermal transducers for a wide range of applications, including water evaporation,^13,17,18^ sanification, desalination,^19−22^ and heat storage.^23−25^ Photothermal efficiencies as high as 90% have been reported for solar thermal collectors using gold or silver water-based nanofluids (approximately 10 nm NPs) with a concentration of just 0.03%v.^26,27^ Alternative plasmonic materials, such as nitrides or carbides, with clear advantages in terms of cost, chemical/thermal stability, hardness, and photon–phonon coupling,^28−32^ over their silver, gold, or graphene counterparts,^35^ remain underexplored for the photothermal conversion in nanofluids, despite their plasmonic nature and good absorption efficiencies.^33,34^

While the nanofluid design has largely focused on the solid fraction, both in terms of overall thermal conductivity and photothermal response,^36,37^ the liquid fraction plays a critical role in heat diffusion and advection. Specifically, the solvent’s thermal diffusivity influences the spatial and temporal propagation of heat across the nanofluid. This aspect holds particular importance in liquid-based energy harvesting, especially in photothermal-driven systems. In this context, thermogalvanic cells stand out as one of the most efficient method for recovering low-grade wasted heat. By creating a higher temperature gradient within a confined section of fluids, two significant advantages can be achieved: (1) enhanced device performance resulting from an increased entropy difference between the hot and cold electrodes and (2) reduced device sizes. Effectively trapping heat within the fluids also presents the opportunity for a direct connection to heat storage systems.^16^ In pyroelectric devices, whose power generation depends on temporal temperature variations, a faster thermal response can also improve the device’s performance.^38^ Moreover, the larger temperature gradient would provoke faster thermodiffusion of the nanoparticles in the fluid, thereby increasing the temporal temperature variation experienced. Consequently, to advance the development of multiphase nanofluids for coupled light harnessing and energy generation systems, a change of paradigm is needed. Depending on the application, optimizing the nanofluid’s temperature profile requires concurrent engineering of the liquid phase’s thermal diffusivity and the solid phase’s optical properties. This approach assumes particular significance in the realm of soft robotics, where space is constrained and the adaptability of liquids holds appeal. Confining heat within a small fluid volume opens avenues for self-powered devices, wherein the thermogalvanic effect could harness energy from ambient radiation to power operations. Finally, it should be mentioned that an emerging class of materials called porous liquids underline the importance of this field.^39^ They have recently been proposed, revealing interesting light-to-heat conversion capability,^40^ along with the possibility of tuning their viscosity,^41^ providing a further degree of freedom to the system design.

**Figure 1:**
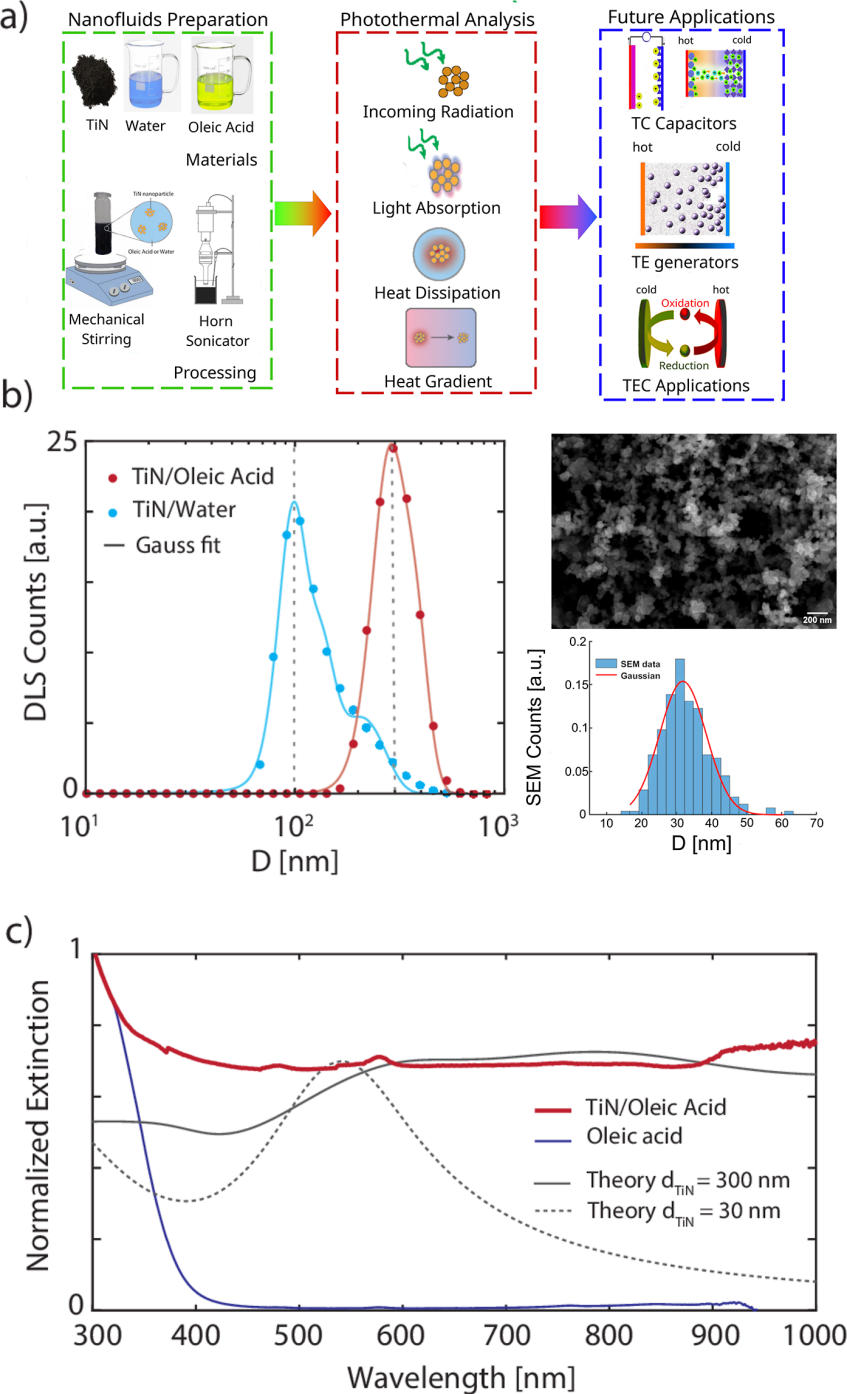
(a) Process of nanofluid realization with schematic representation of the role of solid fraction in photothermal conversion. Insights on the possible application are provided in the final part of the scheme. (b) Nanoparticle size distribution in water and OA obtained with DLS (Nano ZS) for 0.01 wt % titanium nitride concentration (left) and SEM image of the dry powder with the statistical distribution of nanoparticles obtained from the image (right). (c) Extinction coefficient of titanium nitride in OA: red line represents the experimental data (UV-2600i) of the TiN/OA nanofluid and blue line, the bare OA. The black lines are produced using Mie theory for 30 nm particle (dashed) and 300 nm cluster (solid).

In this work, we develop plasmonic nanofluids concurrently exhibiting efficient light absorption and enhanced thermal gradients. This is enabled by the unique combination of an oil-based liquid fraction (oleic acid, OA) and titanium nitride (TiN) nanoclusters (average diameter *D*_NC_ ≈ 300 nm, individual nanoparticles *d*_NP_ ≈ 30 nm, Figure 1b). In particular, thanks to the low heat capacity (*c*_p_), low thermal conductivity (*k*), and high viscosity of OA, in the oil-based nanofluid, we achieved temperature gradients that are ≈ 6 times larger than in the water-based one, reaching up to 15 K/cm under broadband irradiation (xenon lamp, 1000 mW cm^–2^) and 3 K/cm using narrow-band, mild irradiation conditions (LEDs, 40 mW cm^–2^). Additionally, we show that photothermal conversion efficiencies are as high as 89% and can be obtained at a low solid fraction of 0.3 wt %, comparable to that of conventional plasmonic metals. Importantly, we carefully investigate the effects of nanoparticle concentration on light harnessing and heat localization, assessing the trade-off between shorter light penetration depths and increased thermal conductivity. Specifically, we show that the photothermal conversion efficiency increases rapidly from 0.03 to 0.1 wt % solid fraction while preserving a good heat localization. However, beyond 0.1 wt %, the limited penetration depth of light and a likely increase in the thermal conductivity of the fluid hinder further performance gains. Overall, the reported results show new opportunities for designing and engineering plasmonic nanofluids toward liquid-based energy harvesters for liquid robotics applications.

## Results and Discussion

In order to understand the role of the liquid component toward high heat localization and large thermal gradients for stand-alone energy harvesters, we prepared both water-based and OA-based nanofluids by dispersing the TiN nanoparticles into the liquid matrix (Figure 1a). Water represents a straightforward solvent choice due to its availability and intrinsic safety.^42^ Also, because of its high thermal capacity, it is extensively used in nanofluids for heat storage applications.^43^ OA, on the other hand, provides distinct thermal properties while maintaining the characteristics of low cost and biocompatibility. Specifically, OA has lower thermal conductivity compared to water as well as higher dynamic (μ) and kinematic viscosities (ν). Thus, it is expected to enable larger temperature gradients due to increased thermal resistance and limited convective effects.^44−46^ Overall, the main thermo-physical parameters of the chosen solvents are reported in Table 1. We also notice that both fluids are transparent across the visible and near-infrared (NIR) spectra (400–900 nm), with OA exhibiting absorbance in the UV range and water exhibiting IR light absorption.

**Table 1:** Main Thermo-Physical Properties of the Solvents of Interest^47,48^

	*k* [W/(m K)]	ρ [kg/m^3^]	*c*_p_ [J/(kg K)]	μ [Pa s]	ν [m^2^/s]
Water	0.6	998	4182	1 × 10^–3^	1 × 10^–6^
Oleic Acid	0.2	895	2386	26.7 × 10^–3^	39 × 10^–6^

Concerning the solid fraction, the as-received dry powder of TiN nanoparticles was characterized using small-angle X-ray scattering (SAXS), X-ray diffraction (XRD), and high-resolution scanning electron microscopy (SEM), and an average size of *D*_NP_ ≈ 30 nm was estimated (Figures 1b and S1). Since no functionalization is applied to stabilize the nanofluid during the dispersion procedure, the formation of nanoclusters is expected. To determine the size distribution of these, several low-concentration nanofluids (*c* ≈ 0.005 wt %) have been analyzed using dynamic light scattering (DLS). The average distribution is reported in the left panel of Figure 1b for both solvents, where the dashed lines indicate the median.

It can be observed that the oil-based sample shows large nanoclusters (*d*_OA_ ≈ 300 nm) with a good monomodal distribution. On the contrary, polydispersity occurs in water, with a larger fraction of clusters around *d* ≈ 100 nm and a mean *d* ≈ 140 nm. The lower sizes obtained in water are attributed to its polar nature, which contributes to keeping particles separated as observed in other works.^49^ SAXS of a 0.025 wt % oil-based nanofluid further indicates that the average particles size composing the larger agglomerate is around *D*_saxs_ ≈ 28 nm (Figure S1a), in agreement with the SEM estimates of the dry powder. It can be concluded that in the oil-based nanofluid, there are TiN nanoclusters with a dimension of around 300 nm, composed of NPs with a diameter of around 30 nm. It is interesting to notice that the water samples were stable, and no settling occurred even after months of rest. Contrarily, oil samples were settled after 1 month, as can be observed in Figure S2. Considering the time of the experiments, these can be assumed to be stable during measurements, but functionalization is needed to target long-term use.

The information about the NP sizes has been used to reproduce the extinction coefficient measured using a UV–vis spectrometer (Shimadzu UV-2600i). In other words, the analysis of the fluids can be summarized as the initial size of the nanoparticles in the TiN powder was determined directly through electron micrograph inspection. To assess the sizes of the nanoparticles or nanoclusters in the nanofluid, we employed DLS and SAXS. The SAXS image in the Supporting Information clearly indicates the presence of agglomerates, as evidenced by the uptake at low q values. However, determining the dimensions of these clusters from these data is challenging due to the observed uptake. Instead, DLS provided clear evidence of cluster existence as the average dimension was approximately 300 nm. To independently verify the estimated sizes of the nanoparticles and nanoclusters, we relied on the nanofluids’ extinction coefficient and compared it with theoretical estimates based on Mie theory (see the brief summary in SI13). We utilized a MATLAB script that utilizes the data to calculate the extinction coefficient of nanoparticles and nanoclusters with dimensions equal to those estimated via DLS and SAXS. Figure 1c illustrates the excellent agreement between the measured and calculated extinction coefficients, further supporting the accurate analysis of the clustered nature. Here, the spectrum of a low-concentration oil-based nanofluid (*c* = 0.005 wt %) is reported (red solid curve). The strong light absorption at short wavelengths (UV region) can be attributed to the OA (blue solid line). Instead, the extinction coefficient of the TiN nanoclusters, calculated with Mie theory, reproduces well the experimental nanofluid spectrum in the vis–NIR region (gray solid line). In order to calculate the theoretical extinction coefficient of the TiN nanoclusters, it is essential to acquire information regarding the refractive index. This refractive index is related to the permittivity, for which data can be obtained from.^50^Figure S9 presents the representation of this data, along with information on other nitrides and noble metals commonly employed for comparison in similar studies. Finally, the small peak at λ = 580 nm in the nanofluid spectrum can be attributed to the plasmon resonance of the individual TiN nanoparticles forming the nanoclusters. Indeed, the slight red-shift of the observed peak compared to the extinction coefficient of individual *d* = 30 nm TiN nanoparticles calculated with the Mie theory (gray dashed line) can be attributed to the presence of fatty acid chains on the NP surface, as observed in the literature, whose influence is not accounted for in the Mie model.^51^

**Figure 2:**
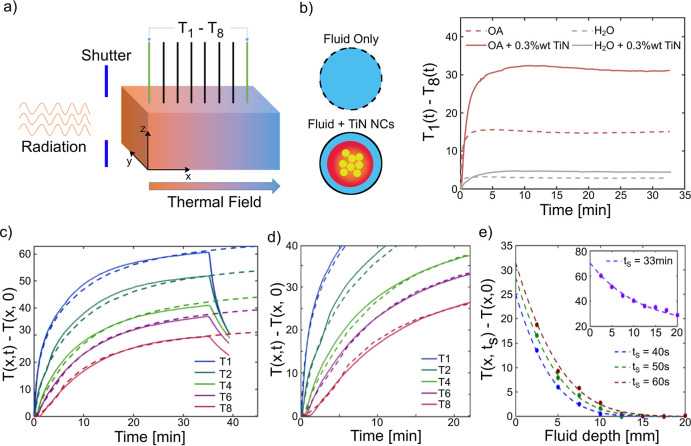
Experimental temperature profile recorded under xenon lamp radiation of OA and water-based titanium nitride nanofluids: (a) schematic representation of the setup used for the characterization. (b) Temperature difference measured across the cell for different solvents and TiN content. (c) Raw measurements (solid lines) and COMSOL model (dashed lines) of the heating profile of 0.3 wt % TiN in OA. (d) Zoom-in image of the early stage of illumination for 0.3 wt % TiN in OA showing the delay in the heating of deeper fluid portion and (e) semi-infinite model analysis of the fluid heating profile at an early stage of illumination with the inset representing the agreement of the steady profile in the fluid with COMSOL model prediction.

To characterize the photothermal response of the plasmonic nanofluids, and in particular, their ability to create a steep temperature gradient, we use a custom 3D printed setup consisting of a 10 × 10 × 20 mm chamber. Up to eight type-k thermocouples are immersed in the nanofluid along the center line of the chamber to measure the temperature as a function of depth and time. The chamber presents a front window of area *A* = 1 cm^2^ for illumination, and a light blocker is used to prevent direct heating of the chamber. A discussion on the error introduced by solvent evaporation due to heating is reported in SI11. A schematic representation of the setup is reported in Figures 2a and S3. We first study the photothermal response of both water- and OA-based nanofluids with *c* = 0.3 wt % of TiN nanoclusters under broadband irradiation using a xenon lamp (λ = 200–2500 nm) whose intensity is adjusted to *I* = 1000 W/m^2^. Upon illumination, the temperature increase of the nanofluids is recorded as a function of time and position until the system reached a steady-state condition. This is characterized by a constant temperature difference between the front and back thermocouples (*T*_1_(*t*) – *T*_8_(*t*) = Δ*T*_1–8_(*t*)) as well as a nearly constant temperature reading for each thermocouple. For the water-based nanofluids without (with) TiN nanoparticles, the temperature difference established across the chamber (Δ*T*_1–8_(*t* = ∞)) does not exceed 3 (5) K (Figure 2b, dashed/solid gray line), corresponding to low-temperature gradients of 1.5 (2.5) K/cm. Indeed, in both samples, with and without solid fraction, we observe a rather uniform temperature increase (see also Figure S4a,b). In the case of oil-only samples, instead, a prominent temperature difference is obtained across the chamber, thanks to the low thermal diffusivity and limited convection (15 K, Figure 2b red dashed curves), leading to an overall temperature gradient of 7.5 K/cm. Most importantly, upon addition of the TiN solid fraction (*c* = 0.3 wt %), the temperature difference across the cell reaches a maximum of 32 K (Figure 2b, red solid line), corresponding to a temperature gradient of 16 K/cm. This represents an increase of about 213% with respect to that of bare OA. Additionally, the largest temperature increase observed in the chamber (thermocouple 1) reaches Δ*T*_1,OA/TiN_ = *T*_1_(*t* = ∞) – *T*_1_(*t* = 0) ≈ 60 K (Figure 2c), which corresponds to ≈50% increase compared to the OA-only sample and largely exceeds the values detected for the water-based nanofluid (Figure S4a,d). This is due to the larger absorption cross-section of the solid fraction in oil matrices (see Figure S4f for *d*_NP_ = 30 nm, where the Mie model has been used to account for different environments surrounding the particles at different sizes and wavelengths). Overall, this shows that strong light absorption of the solid fraction and weak heat transport properties of the fluid matrix can be combined to achieve significant heat localization and large thermal gradients in the nanofluid.

In order to gain a deeper insight into the thermal dynamics of the different nanofluids and understand the role of distinct physical properties, we use a combination of analytical and numerical (COMSOL) modeling. We observe that in the first two min of illumination, the Fourier number is *Fo* ≤ 0.2. Thus, the system can be approximated using a semi-infinite solid with a constant heat flux boundary condition. The analytical solution of the temperature profile is^52^
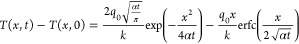
where *q*_0_ is the incoming heat flux, α = *k*/(ρ*c*_p_) is the thermal diffusivity, *k* is the thermal conductivity, and erfc is the complementary error function. For the temperature dependence of the main thermo-physical parameters, we referred to existing literature values using the data to get a function that describes that dependency. Critically, to model the combined characteristics of solid and liquid fractions, colloidal formulas for the specific heat capacity, density, and thermal conductivity must be used.

At the early time scales, convection around the chamber can be neglected, and the incoming heat flux can be estimated from the nanofluid extinction spectrum and the illumination intensity. The results are reported in Figures 2e and S5a for OA and water, respectively, where it can be observed that the trend of experimental data (dot) is well represented by this analytical model (dashed lines).

To gain further insight into both the fast and the slow dynamics of the system, we also developed a 1D COMSOL model. Convection is used as a boundary condition on the front/back surface of the nanofluid chamber, and the convection coefficient increases with time (natural convection). Additionally, we adopt an estimated extinction coefficient to model light absorption as a function of depth in the nanofluid. Overall, using the parameters reported in Table S1, we obtain excellent agreement with the experimental observations across time and positions (Figure 2c for OA and Figure S5b for water). In particular, we can correctly capture the following: (i) the local temperature increase as a function of time; (ii) the earlier temperature increase at the front compared to the back of the chamber due to limited light propagation inside the nanofluid and heat diffusion (see Figures 2d and S6); and (iii) the larger temperature gradient developed across the chamber for the OA-based nanofluid compared to the water-based one. In particular, from the model, we conclude that the system behavior is strongly affected by the light penetration depth, the thermophysical parameter of the liquid matrix (mainly specific heat capacity and thermal diffusivity), and some environmental conditions (*T*, convection around the cell).

Considering that the solid fraction influences both the thermal diffusivity and opacity of the nanofluid, there should exist an optimal concentration that maximizes the heat flux and the resulting temperature gradient. In fact, an increased concentration is expected to entail stronger extinction in the fluid, that is, higher heat generation close to the front surface. At the same time, a higher concentration would enhance heat conduction, leading to a reduced temperature gradient in the fluid. We thus studied the photothermal response of our OA-based nanofluids as a function of concentration. In literature, the solid fraction is typically kept lower than 0.1 wt %, also due to the instabilities rising after this point.^53^ In these experiments, we considered 0.03, 0.06, 0.1, 0.3, and 0.5% wt. Moreover, to minimize the contribution of light absorption in the fluid matrix, we used monochromatic LEDs as illumination sources. These have been chosen in order to cover the UV–vis–NIR part of the electromagnetic spectrum (λ = 375 nm, λ = 420 nm, λ = 530 nm, λ = 625 nm, λ = 780 nm, λ = 940 nm, Figure S7), and the irradiance has been set equal to a constant value of 400 W m^–2^.

**Figure 3:**
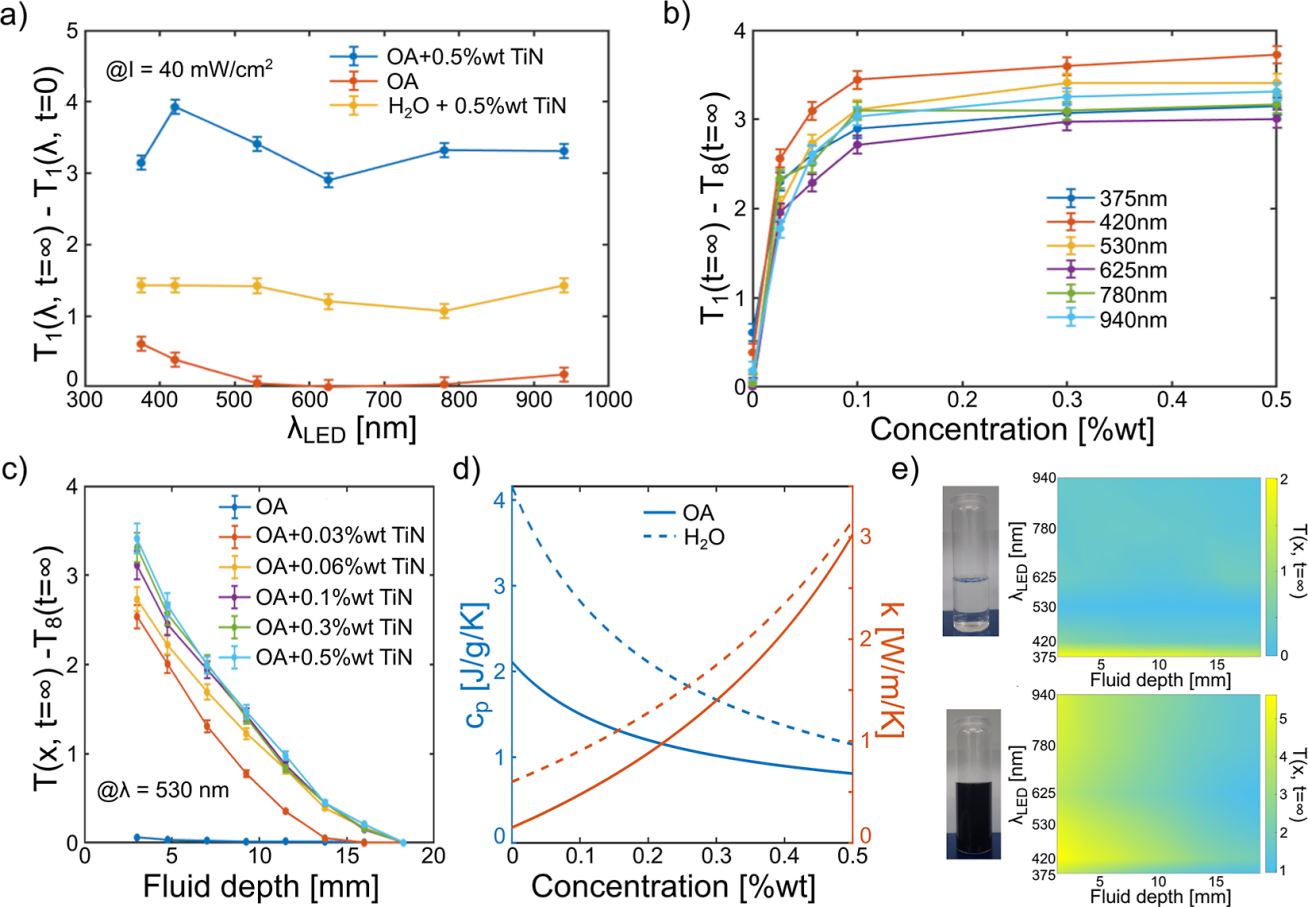
Experimental temperature profile recorded under the LED radiation of OA-based titanium nitride nanofluids. (a) Temperature variation in the first thermocouple recorded as a function of wavelength with comparison to water-based samples. (b) Temperature difference across the fluid as a function of concentration with the inset showing the main thermophysical properties as a function of the solid fraction content. (c) Temperature profile at different concentrations at λ ≈ 530 nm. (d) Specific heat capacity and thermal conductivity of the nanofluids, water (dashed), and oleic acid (solid) as a function of solid fraction. (e) Temperature distribution in 0 and 0.3 wt % titanium nitride in oil-based fluids as function depth and irradiation peak wavelength. On the left, a schematic representation of the setup is reported.

The maximum temperature increase of thermocouple 1 achieved in water- and OA-based nanofluids with concentration *c* = 0.5 wt % is reported in Figure 3a. The steady-state temperature is extrapolated from experimental data, when the plateau is reached. Bare OA characterization is also reported for comparison, showing the slight absorption present in the fluid around λ = 400 nm. It is clear that the OA-based nanofluids outperform the water-based ones at all wavelengths. The observed peak at λ_p_ = 420 nm can be associated with a coupled effect of absorption in both the solid and the liquid fraction. In fact, large TiN clusters can scatter the incoming photons, expanding their path in the liquid matrix. This improves the conversion efficiency and helps confine the heat in the thin layer of fluid. This phenomenon is closely connected to the scattering theory of small particles. It is observed and particularly evident in computations conducted using Mie theory that the scattering cross-section decreases with the nanoparticle dimensions. Conversely, larger dimensions or cluster sizes result in larger scattering, which, according to the theory, predominantly occurs in the forward direction. This implies that the scattered photons have a greater ability to penetrate the fluid. To provide a clearer explanation, Figure S10 has been included in the Supporting Information.

In Figure 3b, the temperature difference between thermocouples 1 and 8 in stationary condition (*T*_1_(*t* = ∞) – *T*_8_(*t* = ∞)) is reported for each LED as a function of TiN concentration. For all wavelengths, we observe a steep increase in the temperature difference (and hence the thermal gradient) upon addition of 0.03 wt % TiN nanoparticle due to the confinement of the heat source otherwise distributed in the fluid. This transition is consistent with previous experiments with the xenon lamp. In fact, in the case of bare fluid, the temperature of all thermocouples was observed to vary immediately at the radiation onset, while a time delay is observed in the presence of a solid fraction (Figure S6). The reason for this phenomenon has to be found in the different distribution of the heating sources. In the case of no particles, the light penetrates deep in the fluid, and the heat is distributed in the fluid. Contrarily, when the particles are present, the light is trapped in the first fluid layers, and successively, the heat is propagated, thanks to conductivity and convection. However, increasing the solid fraction above 0.1 wt % does not lead to further appreciable improvements. This can also be observed in Figure 3c, where the temperature is reported as a function of fluid depth and concentration for λ = 530 nm. Further insights are given observing the concentration dependence of the thermo-physical properties like thermal conductivity or specific heat capacity, as reported in Figure 3d. Interestingly, the higher thermal conductivity of more concentrated samples is compensated by their higher absorption, resulting in an improved temperature gradient (1 K/cm at *c* = 0.03 wt % against 1.8 K/m at *c* = 0.1 wt %). As the concentration increases, we also expect a reduction in the specific heat capacity, meaning that the maximum temperature increase will be higher, given the same energy input. Yet, beyond ≈0.2 wt %, the increase in thermal conductivity is expected to dominate (Figure 3d), and further improvements of the thermal gradient cannot be obtained. Finally, Figure 3e shows the temperature profile as a function of illumination wavelength for the 0.3 wt % TiN/OA sample and the bare OA. The maps of the other compositions can be found in Figure S8. This clearly shows that the liquid and solid fractions work together to capture radiation, affecting the penetration depth across the vis–NIR spectrum.

## Photothermal Efficiency

To estimate the photothermal efficiency of the experimental setup under analysis, the energy stored in the fluid must be quantified. Assuming that the temperature variation occurs only along the depth of the cell (*x*-axis) and treating the *yz* cross-sections as isothermal surfaces, the variation of internal energy for the nonstationary stagnant cell can be expressed as the integral over both space and time of the temperature. The mathematical expression can be found elsewhere.^52^ Considering the limited number of temperature sensors and the finite constant spacing separating them and assuming that the volume around each thermocouple is at a uniform temperature equal to its instantaneous reading, we can compute the experimental change in internal energy as
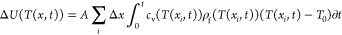
where ρ_f_ indicates the overall nanofluid density accounting for solid and liquid fraction, *c*_v_ the specific heat capacity, and *V*_t_ the total volume of the mixture. From this, the photothermal efficiency of the system can be obtained as the ratio of the energy stored in system Δ*U* and the energy input. This latter is nothing but the radiation power, that is, the irradiance set on the light source *I* impinging on the fluid’s cross-sectional area *A*, multiplied by the radiation time *t*_rad_, translating into
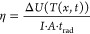


**Figure 4:**
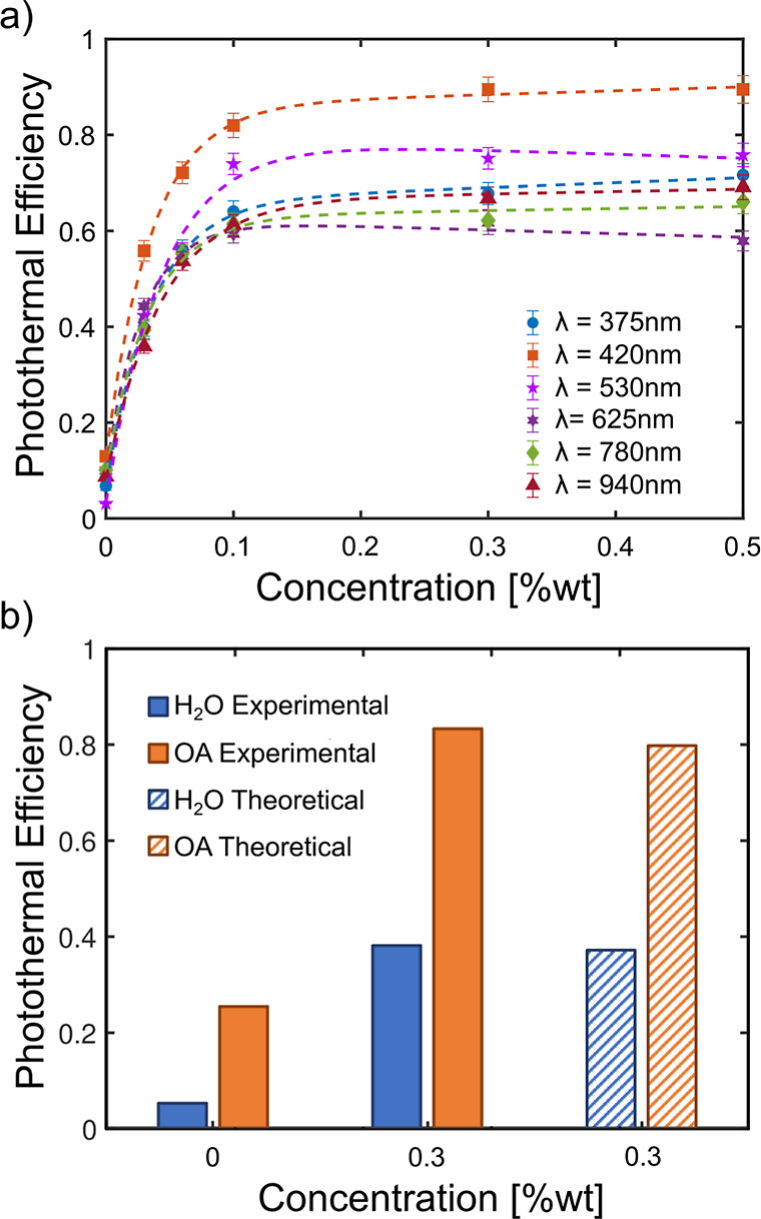
Photothermal efficiency of (a) OA-based nanofluids as a function of titanium nitride concentration using different LED wavelengths; (b) water and OA-based nanofluids with different TiN concentrations under xenon lamp radiation obtained from experimental data (experimental) and comparison to COMSOL predictions (theoretical).

Photothermal efficiency calculations as a function of the concentration and illumination wavelength (LED-based measurements) are reported in Figure 4a. The profile faithfully follows the trend of temperatures, with some slight changes in λ_p_ due to the different times of acquisition. Remarkably, already at 0.03 wt %, the photothermal efficiency is enhanced by ≈ 400% compared to that of the bare fluid, underlining the role of the solid phase for light absorption. These results are in line with the few available literature if the radiation intensity and the different light sources are taken into account.^54−56^ The maximum value of 89% efficiency is reached for the LED λ_p_ = 420 nm at 0.3 wt % sample due to the synergistic work of the solid and liquid fractions. Concerning the xenon lamp irradiation, an efficiency of 83% is calculated from the experimental data. This is in excellent agreement with the estimation based on the COMSOL model (Figure 4b) and confirms the potential of oil-based plasmonic nanofluids for applications where heat localization and large thermal gradients are important, like thermoelectrochemical wasted heat or radiation energy harvesting, with the possibility of exploitation of multiphysical effects, including pyro- and triboelectric ones. The results obtained in our study demonstrate comparability with recent investigations on carbon black-based nanofluids. For instance, Kosinska et al.^14^ conducted an experimental and theoretical analysis, reporting efficiencies as high as η ≈ 90%. Another notable study by Wang et al.^15^ focused on the photothermal activity of iron oxide (Fe_3_ O_4_), where the surface decoration of magnetite with gold led to a 25% enhancement in photothermal conversion. The study achieved efficiencies of up to 80%. Additionally, Zakinyan et al. made an interesting observation regarding the influence of magnetic fields on heat transport in the fluid, providing valuable insights into magnetically controlled liquid memories.^57^ Future advancements could potentially involve the utilization of multiferroic materials, which combine magnetic and ferroelectric properties to explore synergistic effects.

## Conclusions

In conclusion, our photothermal analysis of water- and oil-based plasmonic nanofluids shows that careful choice of the liquid solvent thermal properties and the solid fraction content is critical to achieve high photothermal efficiencies as well as large thermal gradients. In particular, the studied OA/TiN (0.3 wt %) nanofluid exhibits 83% photothermal efficiency and is capable of achieving more than 60 K temperature increase while sustaining a 15 K/cm temperature gradient. The confinement of heat was remarkable already at 0.1 wt %, with interesting dynamics under solar radiation. Furthermore, the proposed approach could be further extended by replacing the oil-based solvent with a liquid permeable matrix with low thermal conductivity (i.e., hydrogels). On the other hand, developing a stable and strong thermal gradient over an irradiated nanofluid induces convective movements within the fluid itself and could be used to directly transform light/heat into active pumping (mechanical energy). Overall, the integration of additional pyro- or triboelectric properties into these plasmonic nanofluids would pave the way to the development of compliant and self-healing liquid-state energy converters for liquid robotics. It should be noticed that amelioration could be achieved also by changing the illumination geometry. In fact, the mechanisms of heat transfer in this framework can be limited to convection and conduction only. In the case of vertical illumination happening on the bottom part of the fluid, it is expected to be a lower thermal gradient since the Rayleigh–Benard convection would be stronger than in the case of lateral illumination. Contrarily, it is likely to have a larger temperature gradient if the illumination happens at the top part of the fluid, where the transport by convection is limited.

## Experimental Section

### Experimental Setup

To obtain information regarding the photothermal efficiency and heat localization in titanium nitride nanofluids, it is necessary to monitor and map the macroscopic temperature distribution throughout the fluid with appropriate spatial and temporal resolution. To achieve this aim, a custom, in-house 3D printed apparatus for photothermal characterization is realized. This is made of polylactic acid by means of a material extrusion additive manufacturing (MEX-AM) approach. For its design, Solid-Worksand CraftWare Pro Slicer software was used. The scheme of the setup is reported in Figure S3. The custom-built setup gives a certain degree of freedom in the design and warrants optical alignment among components. To prevent heating of the fluid container, referred to as the chamber hereinafter, an aperture is designed to limit the illuminated area to the fluid only. The chamber dimensions are 10 mm × 10 mm × 20 mm. A lid was designed to cover the liquid, effectively limiting the convection and evaporation of the fluid. This is equipped with eight type K thermocouples (PeakTech TF-56), where the positive leg is composed of 90% nickel and 10% chromium, while the negative leg is composed of nickel alone. These were chosen due to their suitability for use with liquids, their high resistance to oxidation and corrosion, and their temperature range of −40 to 200 °C with an accuracy of ±2.5%. The thermocouples are positioned in the center of the chamber and aligned at a pitch distance of 2.5 ± 0.5 mm. The error is attributed to the precision of the ruler utilized for positioning the thermocouples rather than the printing accuracy as the latter is considered negligible in comparison to the former. Data were collected by a remotely controlled data acquisition system (DAQ) and subsequently analyzed using MATLAB software. Consider that the error bars depicted in the figures throughout the text are calculated by considering the combined effects of the thermocouples’ accuracy (2.5%), the error associated with their positioning, and the standard deviation of the data. To determine the standard deviation, temperature data are collected in the plateau region, where stationary conditions are achieved, and the standard deviation is computed based on these measurements. Subsequently, the overall error is calculated by taking the square root of the sum of the squared accuracy and the squared standard deviation.

### Dispersion Procedure

The titanium nitride (TiN) NPs used for the experiments were supplied in powder form by PlasmaChem GmbH (Berlin, DE). A sample series of 0.03, 0.06, 0.1, 0.3, and 0.5 wt % concentrations was realized. In order to disperse the particles, each sample was first stirred in the solvent for 2 h vigorously. Subsequently, tip sonication was performed at 40 W to break agglomerates with a duty cycle of 10 s ON and 30 s OFF. To further avoid overheating, which might promote oxidation, a water bath was used. Finally, the nanofluids were stored in a refrigerator (+4 °C) for 24 h. Prior to the actual measurement, the solution was ultrasonicated in a bath to promote dispersion and allowed to thermalize to room temperature. The sedimentation is shown in Figure S2, where the images are acquired in the time range of a month, leaving the nanofluids at complete rest under the hood at room temperature. After a month, the sample appears almost completely settled for all concentrations. The TiN nanocluster relies on steric repulsion for stability in OA and van der Waals repulsion in water, thanks to the polar nature of the water molecules. In the latter, no sedimentation is observed over a period of 2 months, so the stability is already remarkable. Concerning the OA, the sedimentation occurs after a few weeks because of the interaction between the oleic acid chain attached to the nanoparticle’s surface and the ones present in the bulk of the liquid. Here, the stability should be improved using surfactants. In the experiments, it was decided to avoid the functionalization of the particles to avoid contributing to the photothermal effect provoked by the latter, which would be challenging to separate from the ones of titanium nitride nanoparticles. However, sedimentation was considered negligible considering the relatively short time of experiments compared to the sedimentation time, but for practical application, this issue should be addressed.^58^

### Light Source

Initially, the investigation of the temperature profile in the fluid was analyzed using a high-pressure arc lamp (LOT-Oriel GmbH & Co. KG, Germany). This is a xenon lamp with a wavelength range from 200 to 2500 nm and an irradiance of ≈1000 W cm^–2^. This first light source is used to understand the reaction of the system under a situation of high power input. This is needed to understand which properties of the liquid matrix are necessary to localize the heat in a real case scenario, that is, under solar radiation. In the second instance, LEDs were chosen as light sources for two reasons: (i) tunable irradiance, with dependence on both distance and supply voltage and (ii) reduced bandwidth, allowing the investigation of specific excitation. To allow a more careful control, a digital voltage controller coupled with a user interface is used to stabilize the light source, providing the desired power (≈40 mW cm^–2^) and waveform. To cover the UV, visible, and NIR (UV–vis–NIR) parts of the spectra, six LEDs are adopted; the spectra and their deconvolution in two Gaussian distributions are reported in Figure S7, showing peak wavelengths of λ_p_ = 375, 420, 530, 625, 780, and 940 nm.

## Supporting Information Available

SAXS and XRD analysis of bismuth ferrite nanopowder, information about nanofluid stability, detailed aspect of the experimental setup, raw measurement of the photothermal effect under xenon lamp radiation, further analysis using semi-infinite and COMSOL models, details about the transient modification after solid fraction addition, LED spectra, 2D maps of nanofluids heating at different solid fraction loading, real and imaginary permittivity of metals and nitrides, analysis of the nanoparticle cross-section with different sizes and impinging light wavelengths, COMSOL model parameter, and discussion about solvent evaporation and Mie theory (PDF)
