# Correction to “Plasmonic Nanofluids: Enhancing Photothermal Gradients toward Liquid Robots”

**DOI:** 10.1021/acsami.3c15972

**Published:** 2023-11-07

**Authors:** Matteo Bevione, Alessandro Chiolerio, Giulia Tagliabue

In our original paper, in the Acknowledgments section, one sentence is missing. The complete Acknowledgments section is below. This correction does not affect any of the results of the work.

