# Supplementary material for: Plasmonic Nanofluids: Enhancing Photothermal Gradients toward Liquid Robots
Source: ACS Appl Mater Interfaces. 2023 Oct 18;15(43):50106–15. doi: 10.1021/acsami.3c06859 (PMC10623507; doi:10.1021/acsami.3c06859)
Supplement: Supplementary file 1 — am3c06859_si_001.pdf [file am3c06859_si_001.pdf]

SUPPORTING INFORMATION

Plasmonic Nanofluids: Enhancing Photothermal Gradients Towards Liquid Robots

Matteo Bevione[1,2], Alessandro Chiolerio[3], and Giulia Tagliabue[2, *]

[1] *Empa - Swiss Federal Laboratories for Materials Science and Technology, Ueberlandstrasse 129, 8600 Duebendorf*

[2] *École Polytechnique Fédérale de Lausanne, Laboratory of Nanoscience for Energy Technology (LNET), Rte Cantonale, 1015 Lausanne*

[3] *Center for Converging Technologies, Soft Bioinspired Robotics, Istituto Italiano di Tecnologia, Via Morego 30, 16163 Genova*

Supplementary Information 1 – SAXS Measurements

Fig. S1a reports small angle x-ray scattering of a $0.025\backslash\%wt$ oil based nanofluid. Here the scattering provoked by the oil matrix is accounted as background and removed. The raw measurement is shown while the line integration shows an inflection around $q = 2.2A$. This indicates that the average particles size composing the larger agglomerate, is around $d_{\{saxs\}} \approx 28nm$, in agreement with the SEM observation (inset figure). Moreover, the uptake at low q value is a clear indication that agglomeration occurs. However, we would need lower q value to estimate the agglomerate dimension since the change of steepness in the curve cannot be determined from these data.

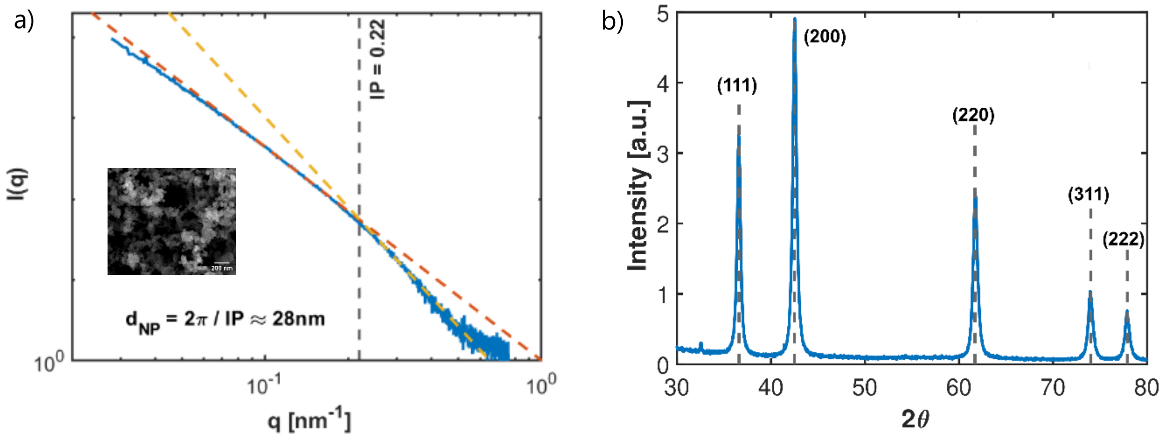

Figure S1: a) Small angle x-ray scattering of 0.025\%wt oil based nanofluid. b) XRD of the as-synthesized titanium nitride nanopowder.

Corresponding author email: giulia.tagliabue@epfl.ch

Supplementary Information 2 – Nanofluid stability

Fig. S2 (a to d) reports photographs of the oleic acid-based nanofluids at different concentration left to rest over a period of 1 month to show their stability.

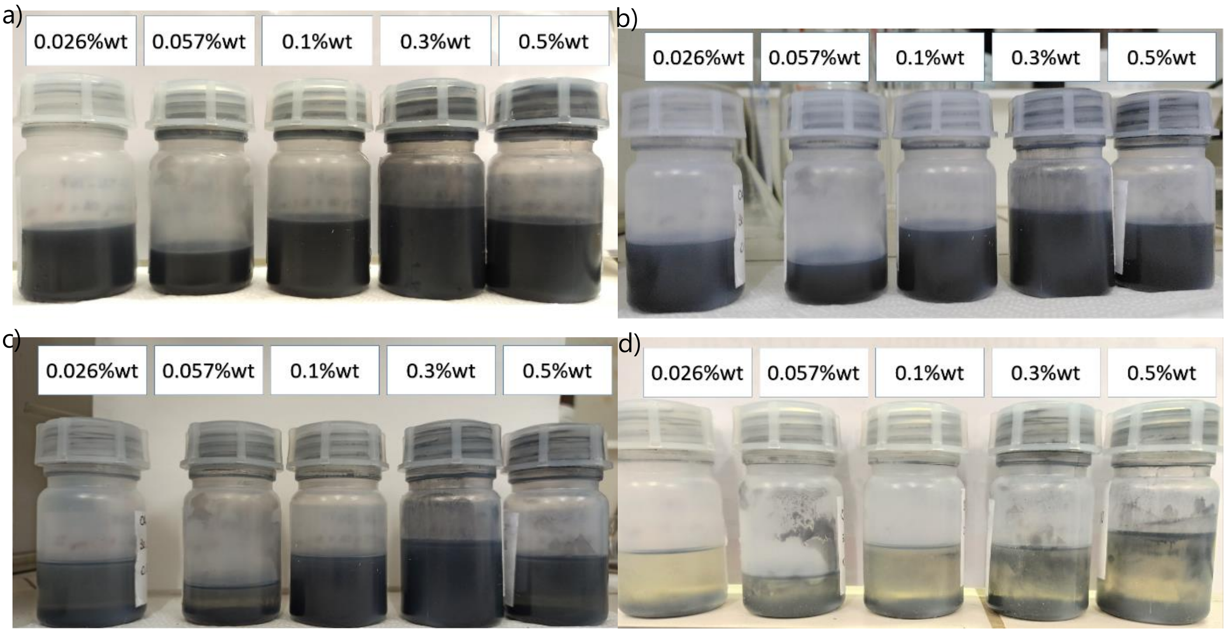

Figure S2: Sedimentation behavior of oleic acid based titanium nitride nanofluids at different concentration. Picture taken after a) 1 day, b) 1 week, c) 2 weeks and d) 1 month of rest under the hood.

Corresponding author email: giulia.tagliabue@epfl.ch

Supplementary Information 3 – Experimental Set-up

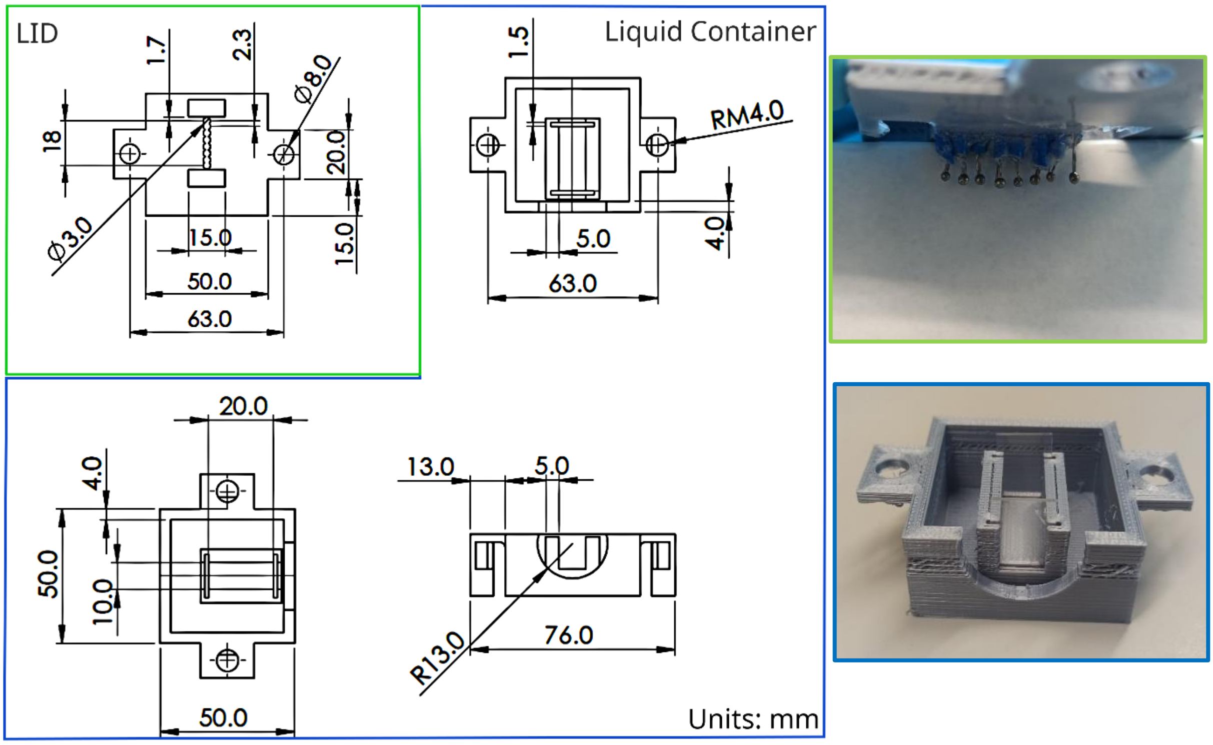

Figure S3: Setup used for the characterization of the nanofluids. On the left the quotes and designed made using Solidworks. On the right the final product, 3D printed using FDM method in PLA.

Corresponding author email: giulia.tagliabue@epfl.ch

Supplementary Information 4 – Raw measurements nanofluids under xenon lamp radiation

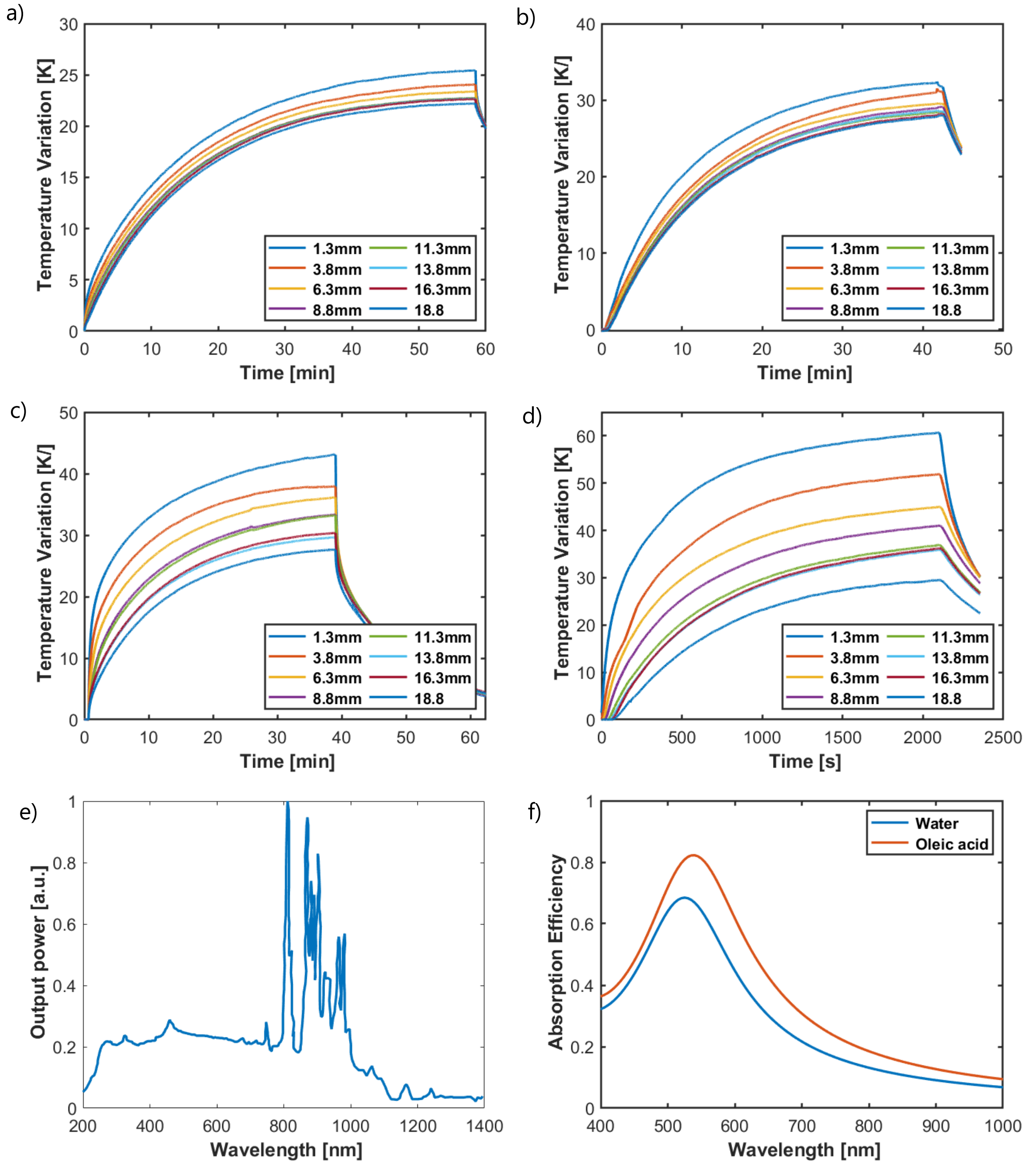

Figure S4: Raw measurement of the heating in a) water, b) water + 0.3%wt TiN, c) oleic acid and d) oleic acid + 0.3%wt TiN under xenon lamp radiation; e) Xenon arc lamp spectra; f) Absorption efficiency calculated using Mie model of single spherical titanium nitride in water and oleic acid.

Corresponding author email: giulia.tagliabue@epfl.ch

Supplementary Information 5 – Semi-infinite and COMSOL model

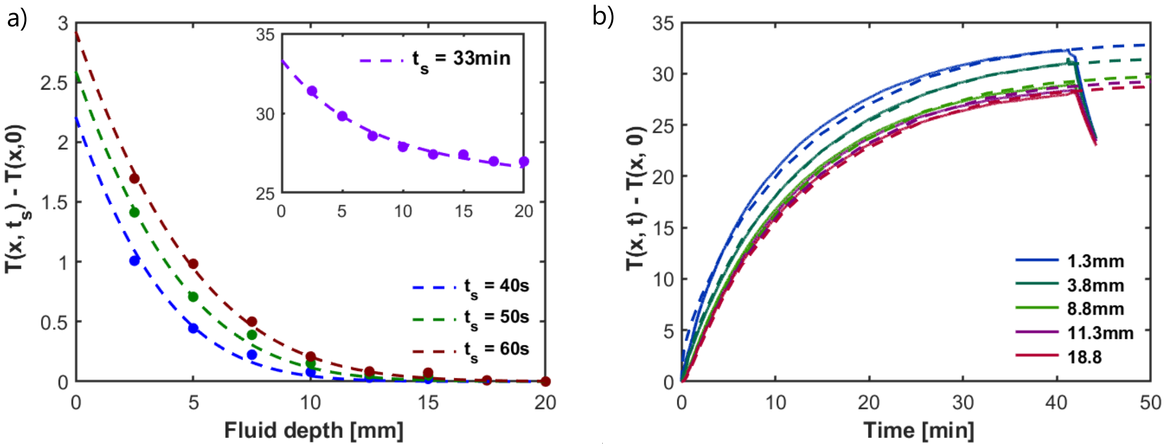

Figure S5: a) Semi-infinite model analysis of the temperature profile in water + 0.3%wt titanium nitride under xenon lamp radiation in the early stages of illumination. b) Temperature profile measured over time experimentally (solid lines) and modeled with COMSOL (dashed lines).

Corresponding author email: giulia.tagliabue@epfl.ch

Supplementary Information 6 – Transient raw measurements under xenon lamp radiation

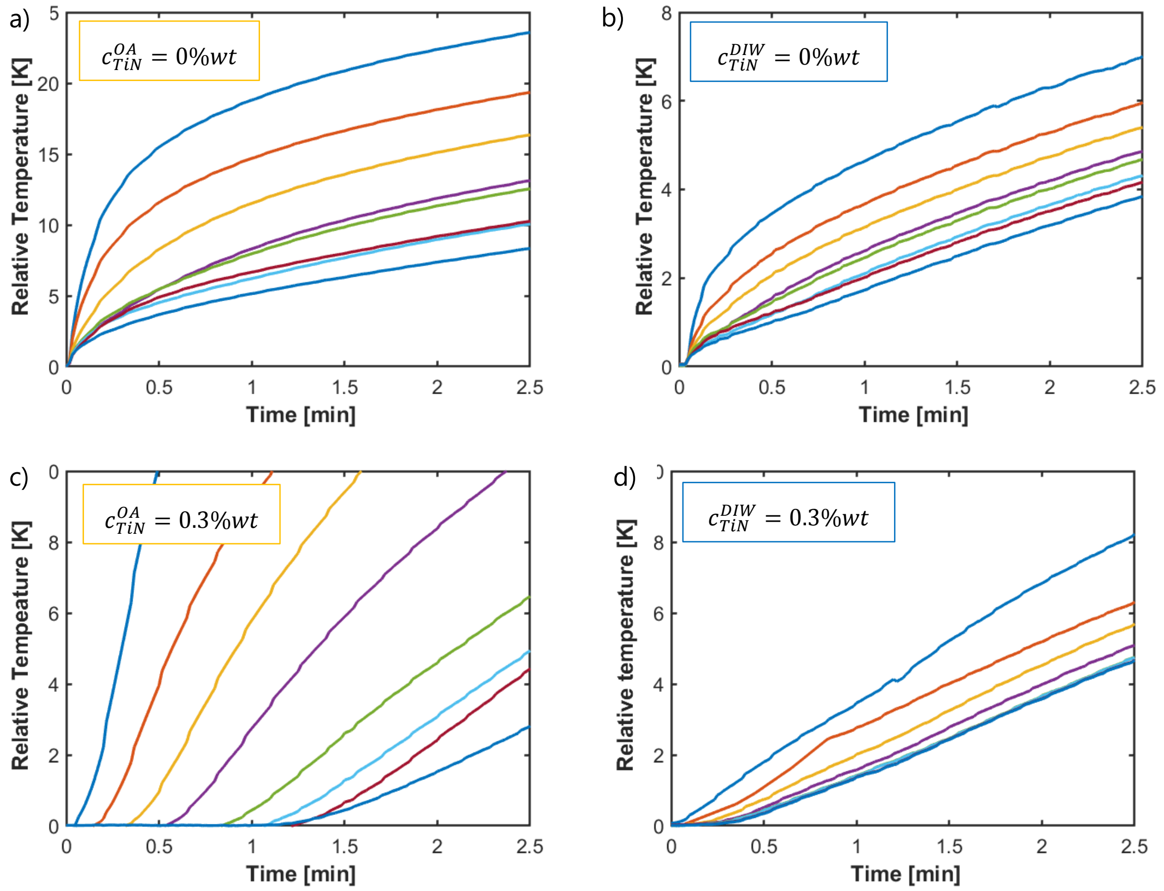

Figure S6: Zoom of the raw measurement of oleic acid and water based fluids (a,b) and with solid content of 0.3%wt titanium nitride (c, d) at the early stages of xenon lamp irradiation to show the different behavior due to the presence of solid fraction in the deeper portion of fluids (from distributed to confined heat source).

Supplementary Information 7 – LEDs Emissions Profile

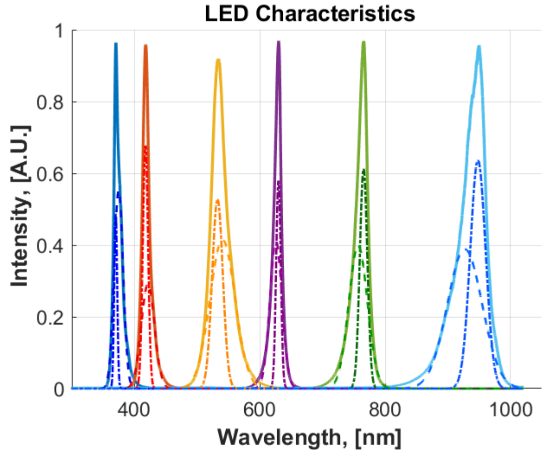

Figure S7: Spectra of the LEDs used for the experiments.

Corresponding author email: giulia.tagliabue@epfl.ch

Supplementary Information 8 – Heating map of OA-based nanofluids

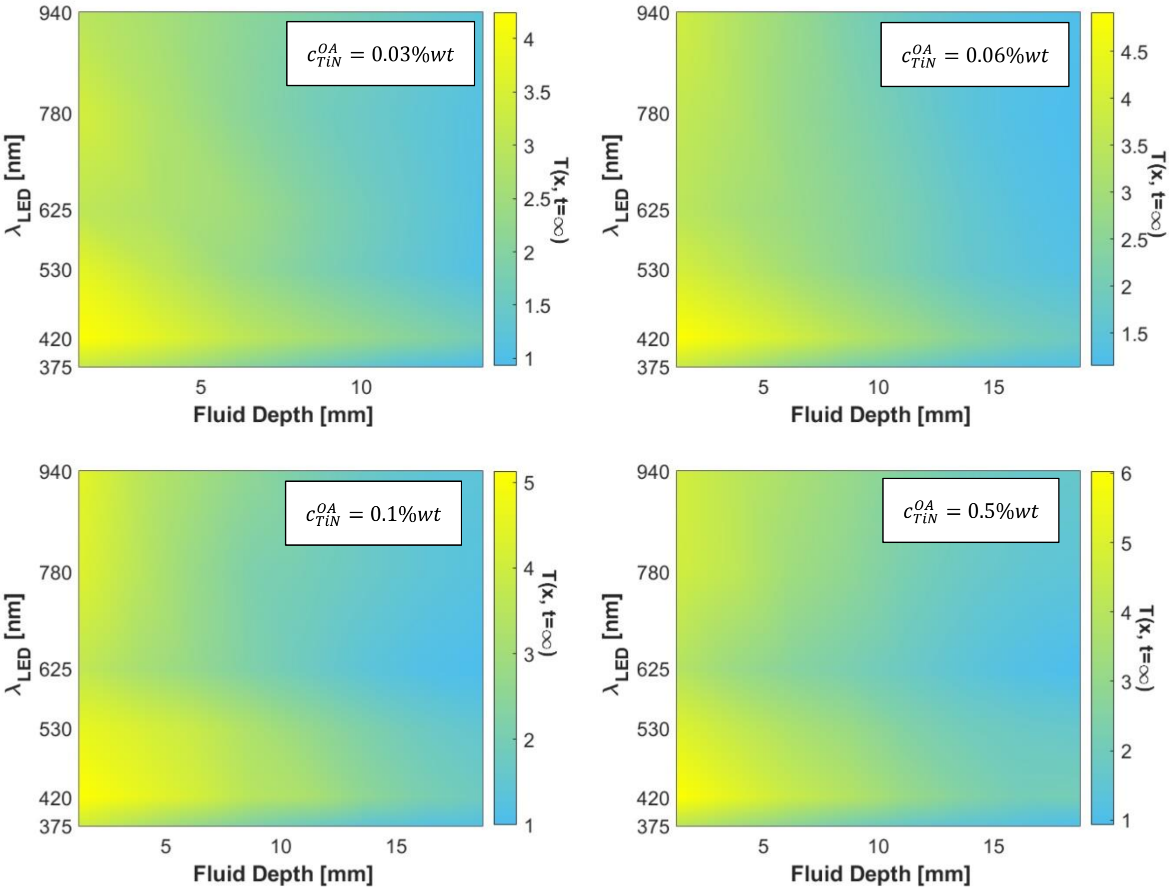

Figure S8: 2D map of oleic acid-based fluids under different LEDs radiation as a function of depth in the fluid.

Supplementary Information 9 – Permittivity of metals and nitrides

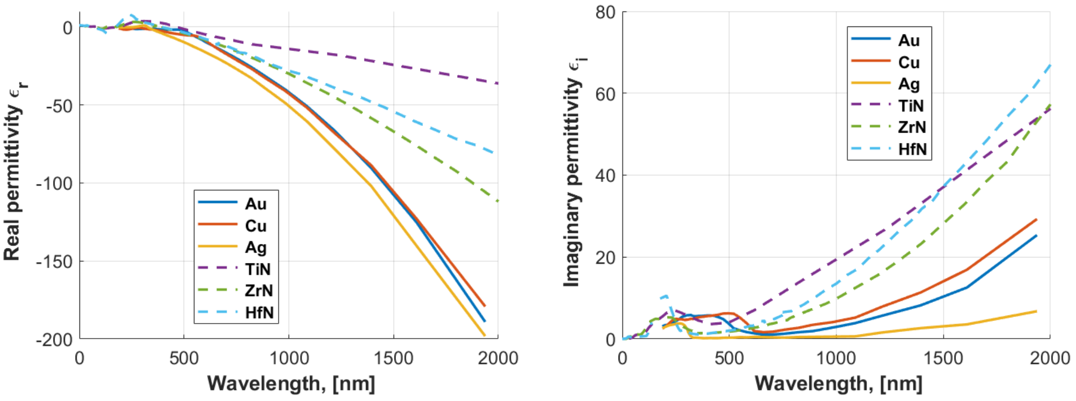

Figure S9: (left) Real and (right) Imaginary part of titanium nitride permittivity. This is reported along with other nitrides and noble metals for sake of comparison.

Corresponding author email: giulia.tagliabue@epfl.ch

Supplementary Information 10 – TiN nanoparticles cross sections

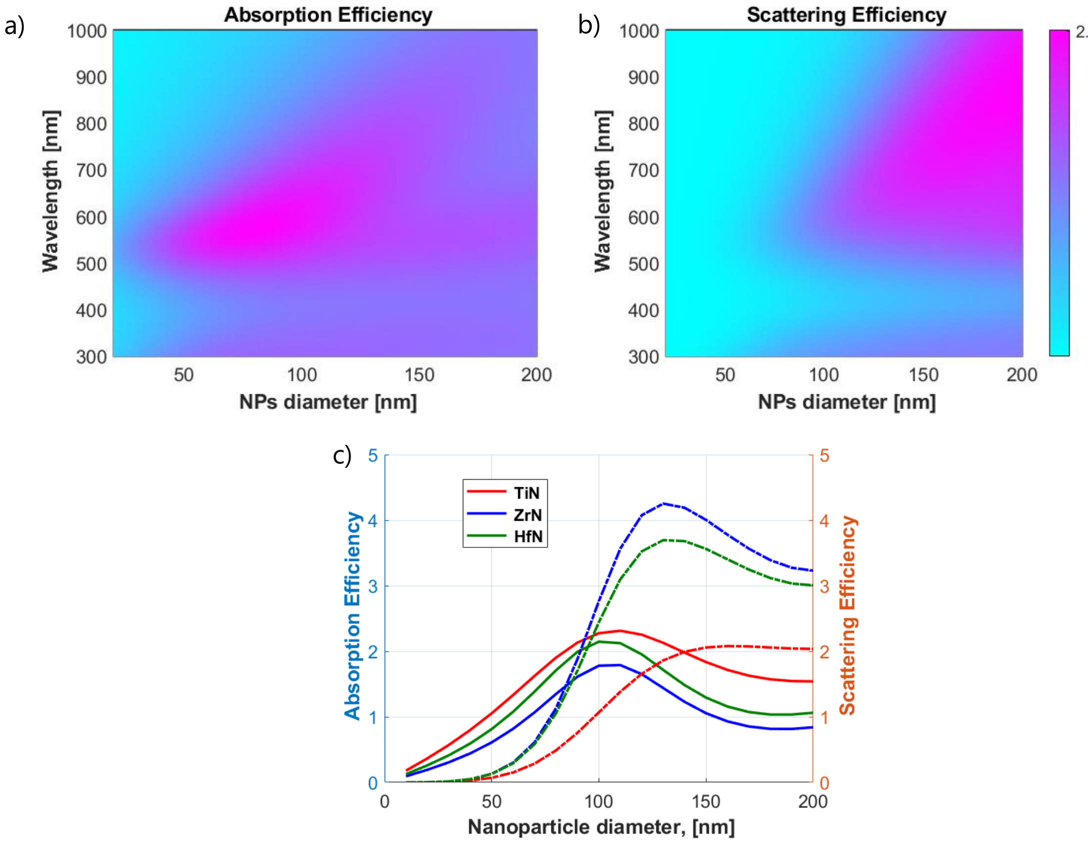

Figure S10: a) Absorption and b) Scattering of TiN nanoparticles with size in relation with incoming radiation. C) Efficiencies of nitrides vs diameter at $\lambda = 450nm$.

Supplementary Information 11 – Parameters used in COMSOL Model.

Table S1: Main parameter used in COMSOL simulation under xenon lamp radiation.

Name	Oleic acid	Water	Notes	Name	Oleic acid	Water	Notes
I_0	1000 $[W/m^2]$	1000 $[W/m^2]$	Illumination Intensity	c_{mu}	36.18 $[Pa \cdot s]$	1.177 $[Pa \cdot s]$	Dynamic viscosity of solvent 2
α	20000 $[1/m]$	15000 $[1/m]$	Absorption coefficient	d_{mu}	-0.0061 $[1/K]$	-0.0041 $[1/K]$	Dynamic viscosity of solvent slope 1
h_{conv}	10.2 $[W/m^2/K]$	13 $[W/m^2$ $/K]$	Convection coefficient	$a_{c_{P_{TiN}}}$	722.9 $[J/kg/K]$	722.9 $[J/kg/K]$	Specific heat titanium nitride parameter

Corresponding author email: giulia.tagliabue@epfl.ch

$h_{convback}$	12 $[W/m^2/K]$	1 $[W/m^2/K]$	Convection coefficient back side	$b_{c_{p_{TiN}}}$	0.0001601 $[1/K]$	0.0001601 $[1/K]$	Specific heat titanium nitride parameter
p_{ref}	1 $[atm]$	1 $[atm]$	Atmospheric pressure	$c_{c_{p_{TiN}}}$	-1808 $[J/kg/K]$	-1808 $[J/kg/K]$	Specific heat titanium nitride parameter
ρ_1	-0.6714 $[kg/m^3/K]$	$-3.54e^{-3}$ $[kg/m^3/K]$	Slope of the density vs temperature	$d_{c_{p_{TiN}}}$	-0.0082 $[1/K]$	-0.0082 $[1/K]$	Specific heat titanium nitride parameter
ρ_2	1090 $[kg/m^3]$	1862 $[kg/m^3]$	Intercept of the density vs temperature	ρ_{TiN}	5240 $[kg/m^3]$	5240 $[kg/m^3]$	Titanium nitride bulk density
a_{mu}	$2.735e+07$ $[Pa \cdot s]$	$3.073e^4$ $[Pa \cdot s]$	Dynamic viscosity of solvent	$a_{c_{poa}}$	3164 $[J/kg/K]$	4186 $[J/kg/K]$	Specific heat titanium of liquid
b_{mu}	-0.04689 $[1/K]$	-0.03664 $[1/K]$	Dynamic viscosity of solvent slope 1	$b_{c_{poa}}$	11.5 $[J/kg/K^2]$	-	Specific heat titanium of liquid coefficient.
φ	0.003	0.003	volume ratio of titanium nitride	k_{TiN}	$29e^{-3}$ $[W/(m \cdot K)]$	$29e^{-3}$ $[W/(m \cdot K)]$	TiN thermal conductivity at 323K

Supplementary Information 12 – Solvent Evaporation

Water evaporation depends on several factors among which are the area of the tank (A), liquid temperature (T), pressure (p), velocity of air above the free surface of the liquid (v), maximum saturation humidity ratio in the air (x_s), humidity ratio for air (x), and evaporation heat (enthalpy) of the liquid. Among these parameters, the ones contributing the most to limiting evaporation are the free surface and the air temperature and speed. Considering the experimental setup, the cell is covered with a lid and the liquid tank is completely filled, limiting the air speed flowing above the cell. Moreover, the area considered is strongly limited since the only possible points of contact between air and water are found in the holes hosting the thermocouples. However, for the sake of simplicity and overestimation, let us consider the lid as not present so that the surface of the water in contact with air is the largest possible. The rate of water evaporation (g_s) expressed in kg/s^{-1} from a tank can be expressed according to

$$g_s = \frac{(25 + 19v)A(x_s - x)}{3600}$$

Based on the Ideal Gas Law the humidity ratio can be expressed as

$$x = 0.62198\, p_{ws} / (p_a - p_{ws})$$

where $p_{ws} = \frac{e^{77.345 + 0.0057\,T - \frac{7235}{T}}}{T^{8.2}}$ is the saturated pressure of water vapor in moist air (Pa, psi) and p_a is atmospheric pressure of moist air. Considering the maximum temperature reached in the experiments and substituting the values corresponding to this latter we can estimate the evaporation rate. Due to

Corresponding author email: giulia.tagliabue@epfl.ch

the lack of forced cooling, the natural air convection velocity is assumed to be 1m/s. According to this, the estimated loss of water mass in an hour is $\approx 250\mu g$ representing 0.025% of the water mass present in the tank. Notice that this value is highly overestimated since the water tank is not kept at the maximum temperature for so much time and we do not have such a large area exposed to air. Moreover, the thermocouples are dipped in the bulk of the water so the temperature is measured away from its free surface. All considered the mass loss could affect the measurements but not in a sensitive way, in other words, the error provoked by such evaporation is small compared to measurement errors.

Supplementary Information 13 – Mie Theory

The data about nanoclusters sizes were obtained using dynamic light scattering (DLS Nano ZS). Successively, this information has been used to calculate the extinction coefficient according to Mie's theory. This theory is well established and the MATLAB code used is based on this book. We can summarize the procedure as follow:

- Use spherical coordinates to describe the plane wave.
- Expansion of the electromagnetic field inside and outside the particle;
- Reduce them into series expansion: the coefficients are obtained for the field inside a particle and for the scattering field;
- The coefficients of scattering Q_s and extinction Q_e and absorption efficiencies Q_a, are calculated by integrating the Pointing vector with respect to angle and space variables.

This process ends in an infinite series for the scattering C_s and extinction C_e cross-section, namely:

$$C_{sca} = \frac{2\pi}{\kappa^2} \sum_{n=1}^{\infty} (2n+1)(|a_n|^2 + |b_n|^2)$$

$$C_{ext} = \frac{2\pi}{\kappa^2} \sum_{n=1}^{\infty} (2n+1) Re(a_n + b_n)$$

where the coefficients of the series are:

$$a_n = \frac{\mu\, m^2 j_n(mx)[xj_n(x)]' - \mu_1\, j_n(x)[mxj_n(mx)]'}{\mu\, m^2 j_n(mx)[xh_n^1(x)]' - \mu_1 h_n^1(x)[mxj_n(mx)]'}$$

$$a_n = \frac{\mu_1\, j_n(mx)[xj_n(x)]' - \mu\, j_n(x)[mxj_n(mx)]'}{\mu_1\, j_n(mx)[xh_n^1(x)]' - \mu h_n^1(x)[mxj_n(mx)]'}$$

with the prime indicating the derivative w.r.t. the argument in parentheses, m is the ratio among the particle's refractive index n_p and the medium n_m, x is the size parameter, i.e. the product of wavevector k and r, the particle's radius and j_n^1, h_n^1 are the Bessel and Hankel functions of the first kind, respectively.

Corresponding author email: giulia.tagliabue@epfl.ch
